# Barrier Coverage for 3D Camera Sensor Networks

**DOI:** 10.3390/s17081771

**Published:** 2017-08-03

**Authors:** Pengju Si, Chengdong Wu, Yunzhou Zhang, Zixi Jia, Peng Ji, Hao Chu

**Affiliations:** 1 College of Information Science and Engineering, Northeastern University, Shenyang 110819, China; 2 Faculty of Robot Science and Engineering, Northeastern University, Shenyang 110819, China; wuchengdong@ise.neu.edu.cn (C.W.); zhangyunzhou@ise.neu.edu.cn (Y.Z.); jiazixi@ise.neu.edu.cn (Z.J.); jipeng@ise.neu.edu.cn (P.J.); chuhao@ise.neu.edu.cn (H.C.)

**Keywords:** barrier coverage, camera sensor networks, resolution, 3D sensing model

## Abstract

Barrier coverage, an important research area with respect to camera sensor networks, consists of a number of camera sensors to detect intruders that pass through the barrier area. Existing works on barrier coverage such as local face-view barrier coverage and full-view barrier coverage typically assume that each intruder is considered as a point. However, the crucial feature (e.g., size) of the intruder should be taken into account in the real-world applications. In this paper, we propose a realistic resolution criterion based on a three-dimensional (3D) sensing model of a camera sensor for capturing the intruder’s face. Based on the new resolution criterion, we study the barrier coverage of a feasible deployment strategy in camera sensor networks. Performance results demonstrate that our barrier coverage with more practical considerations is capable of providing a desirable surveillance level. Moreover, compared with local face-view barrier coverage and full-view barrier coverage, our barrier coverage is more reasonable and closer to reality. To the best of our knowledge, our work is the first to propose barrier coverage for 3D camera sensor networks.

## 1. Introduction

Barrier coverage of wireless sensor networks is a fundamental issue where the objective is to construct a long narrow barrier belt area of sensors to detect intruders that attempt to cross the deployed region. Barrier coverage serves a variety of applications such as national border control, critical resource protection, security surveillance, and intruder detection [1]. Due to its unique requirements, the barrier coverage of a wireless sensor networks exhibits different characteristics and calls for different design considerations as compared to other coverage measures such as area coverage [2,3] and target coverage [4,5,6]. The issues of barrier coverage have been discussed in traditional scalar sensor networks [7,8] and camera sensor networks [9,10], respectively.

Previous studies on barrier coverage mainly focused on traditional scalar sensor networks, in which the sensing range of a sensor is often modeled as a disk and an object is said to be covered or detected by a sensor if it is within the sensing range of the sensor [11,12]. Compared with traditional scalar sensors, camera sensors can provide much richer information about the environment in the forms of images or videos and hence have huge potential in applications.

For barrier coverage, how to use sensor nodes to meet the mission requirements for monitoring tasks is still an open issue. However, the barrier coverage of camera sensors is more complicated than traditional barrier coverage. In fact, one fundamental difference between camera sensors and traditional scalar sensors in coverage is that camera sensors may generate different views of the same object if they are from different viewpoints [1]. The content of barrier coverage in camera sensor networks focuses on the moving targets (e.g., persons and vehicles) through the barrier area. However, in most of the previous models, targets were simply considered as points. The disadvantage of these models is that some physical features (e.g., size, shape and color) of targets are neglected.

In recent years, more and more concepts of barrier coverage have been developed to meet the demands of the moving target. A target is said to be full-view covered if there is always a camera sensor to cover it no matter which direction it faces and if the camera sensor’s viewing direction is sufficiently close to the target’s facing direction [13]. In [14], the authors proposed local face-view barrier coverage, a novel concept that achieves statistical barrier coverage in camera sensor networks leveraging intruders’ trajectory lengths along the barrier and head rotation angles. The above two concepts take into account the effective angle of the camera sensor’s viewing direction and the intruder’s facing direction. However, both full-view barrier coverage and local face-view barrier coverage only consider the sector sensing model, which is a two-dimensional (2D) model. Not only should the effective angle in the horizontal direction be utilized, but also the effective angle in the vertical direction should be taken into account in the real application. For example, the reason for full-view barrier coverage is that the facing direction of an intruder and the viewing direction of a camera sensor may result in a certain angle in the horizontal direction when the intruder is crossing the sensing area of the camera sensor. Similarly, the reason for local face-view barrier coverage is that the intruder’s head may rotate either to the left or to the right within a certain angle. However, for most real-world applications, camera sensors are deployed within a certain height from the ground, which can result in an angle in the vertical direction between the intruder’s facing direction and the viewing direction of one camera sensor. Besides, the intruder’s head can also rotate up and down. Then, we should consider the three-dimensional (3D) sensing model of camera sensors to construct real barrier coverage.

**Figure 1:**
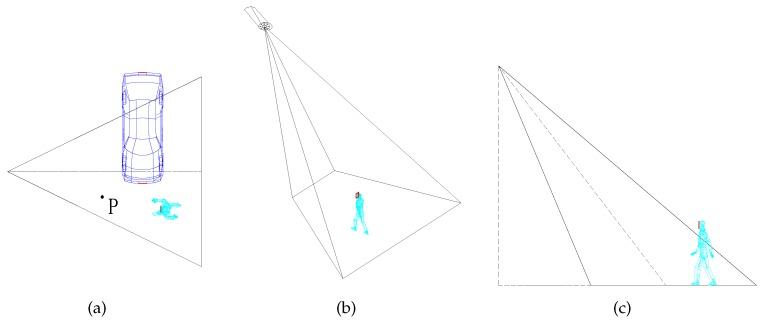
(**a**) There are three different types of targets (a point P, a car and a person) in the sensing area of the camera sensor. Obviously, it is not appropriate to use points to express different targets. Furthermore, the camera sensor cannot capture the license plate number of a car because of the camera sensor’s viewing direction; (**b**) A person is seemingly captured by the camera sensor when he is walking in the sensing area of the camera sensor; (**c**) However, the camera sensor cannot recognize the walking person because the person’s face is not in the sensing area of the camera sensor.

Camera sensors can capture a rectangular image of the intruder in camera sensor networks. In the image, what we are concerned with is the region of interest (ROI), which depends on the kind of monitoring task. For example, when a camera sensor captures a person, the ROI is usually the person’s face, rather than the person’s back. On the road crossing or the parking entrances, the ROI is usually the license plate number of the car instead of the brand. Figure 1a shows an example of different types of targets (a point P, a car and a person) in the sensing area of the camera sensor. As we can see from the figure, the point P can be effectively detected by the camera sensor. However, the realistic targets may not be accomplished with desirable performance. Only a portion of the car can be captured by the camera sensor because the other portion is not in the sensing range of the camera sensor. Since the angle between the car’s facing direction and the camera sensor’s viewing direction is too large, the camera sensor cannot read the license plate number of the car. As depicted in Figure 1a,b, the person is seemingly captured by the camera sensor when he is walking in the sensing area of the camera sensor. However, as illustrated in Figure 1c, the camera sensor cannot recognize the walking person because the person’s face is not in the sensing area of the camera sensor. Obviously, it is not appropriate to use points to express different types of targets when we consider the ROI (e.g., the license plate number of the car and the person’s face). Therefore, to maintain high-level surveillance quality, the types of targets should be taken into account in to how to achieve the barrier coverage.

Furthermore, for the task with high resolution, even though the ROI of a target is located in the sensing area of the camera sensor, the target cannot be effectively captured when the target is detected with low resolution by the camera sensor. As shown in Figure 1c, the person’s face will be detected by the camera sensor when the person is walking straight into the sensing area of the camera sensor. However, this does not mean that the image of the person’s face is with high resolution. All factors, including the resolution of camera sensor, the size of the ROI, the angle and the distance between the camera sensor’s viewing direction and the person’s facing direction can influence the resolution of the ROI. In addition, the desired resolution corresponds to the monitoring task. For example, in the application of face recognition, the required resolution for the identifying gender is quite different from that identifying who it is. Only with the desired resolution of the ROI can the target be effectively detected and captured by the camera sensors.

Hence, with these observations, we study the barrier coverage problem with a 3D sensing model of camera sensors. Furthermore, we propose a new resolution criterion based on 3D sensing model of camera sensors for capturing intruder’s face (or the ROI). Our contributions in this paper are summarized as follows:

- We propose a new resolution criterion for capturing the ROI of target. Instead of considering the target as a point, the ROI of target should be considered as a rectangular area. Given the requirements of task, the effective resolution sensing model is related to α and β. α and β are the effective horizontal angle and the effective vertical angle between the camera sensor’s viewing direction and the target’s facing direction, respectively.
- We study the barrier coverage according to the tasks’ requirements with our resolution criterion in camera sensor networks. First, we analyse the width of barrier area, the distance between the camera sensors, and the coverage probability of barrier coverage. Second, through the extensive evaluation, the results illustrate that our proposed concept was correct. Compared with full-view barrier coverage and local face-view barrier coverage, the required number of camera sensors in the barrier coverage using 3D sensing model was more reasonable.

The rest of this paper is organized as follows. Section 2 highlights the related work. Section 3 formulates 3D sensing model based on ROI. Section 4 studies barrier coverage based on the 3D sensing model. Section 5 evaluations of our theoretical results. Section 6 discusses of the robustness and fault tolerance of our barrier coverage. The paper concludes with Section 7.

## 2. Related Work

Sensor coverage, one of the fundamental problems of sensor networks, tries to answer the questions about the quality of sensing (surveillance) that a particular sensor networks provides [15]. Depending on the type of application, coverage problems can be divided into point coverage [5], area coverage [16], and barrier coverage [17]. A major goal is to detect intruders as they cross a border or as they penetrate a protected area. This type of coverage is referred to as barrier coverage, where the sensors form a barrier for the intruder, and sensor nodes deployed in a region is said to provide barrier coverage if and only if the intruder’s crossing path intersects the detection range of at least one sensor [7,18]. According to various criteria, barrier coverage can be divided into strong and weak barrier coverage, single and K-barrier coverage, respectively. Liu et al. [19] introduced strong and weak barrier coverage problems of wireless sensor networks using scalar nodes. Weak barrier coverage only guarantees detection of intruders moving along congruent paths. In contrast, strong barrier coverage guarantees detection of intruders no matter what crossing paths they take. Besides, the K-barrier coverage problem, whether all the intruder’s crossing paths through the barrier coverage area are K-covered and that a crossing path is said to be K-covered if it can be covered by at least K distinct sensors was discussed in [20].

Coverage problems with uncertain properties, including probabilistic and directional sensing models, should be considered in practical applications [21]. In the traditional sensor networks [15,22], the authors justified a target is effective for sensing by a sensor node when the target is in the sensing area of the sensor, which is referred to as an omin-directional deterministic sensing model. Ahmed et al. [23] investigated the coverage issues in wireless sensor networks based on probabilistic coverage and proposed a distributed probabilistic coverage algorithm. Probabilistic sensing is valid for certain kinds of sensors (e.g., temperature sensors, acoustic sensors and seismic sensors), where the signal strength decays with the distance from the source [24]. In order to detect an intruder, Onur et al. proposed a probabilistic detection model with false alarm rate in [25].

Compared with traditional scalar sensors, camera sensors provide much richer information of the environment in the forms of images or videos and hence promise huge potential in applications [26]. Furthermore, most previous studies in camera sensor networks mainly took into account the deterministic sensing models of camera sensors, such as sector [27] and trapezoid sensing models [28]. Based on the directional sensing model, Zhang et al. [29] studied the weak and strong barrier coverage problems of directional sensor nodes, and they presented three methods to solve the maximum directional sensor barrier problem (MDSBP). Wang et al. discussed the K-barrier coverage problem with minimum number of mobile sensors required and the maximum number of barriers that can be formed given the stationary and mobile sensors in [30]. Furthermore, in [31], they studied the barrier problem with directional and omni-directional sensing models, respectively.

Furthermore, since camera sensors can achieve more spatial information than traditional scalar sensors, scholars began to study a 3D sensing model for camera sensors.

Ma et al. [32] proposed more realistic 3D sensing model of the camera sensor to achieve area coverage. Munishwar et al. [33] defined a new problem of deriving the optimal set of field-of-view (FoV) to be considered by PTZ camera sensors. The performance of PTZ coverage algorithms can be substantially increased by reducing the number of candidate FoVs for each camera sensor. Rather than focusing on camera sensor parameters, they focused on the group of targets covered. Barr et al. [34] proved that there is no strong barrier coverage in a large 3D underwater scalar sensor networks. However, to the best of our knowledge, there is no existing work to study barrier coverage with 3D sensing model of camera sensors.

For better consideration of the intrinsic property of camera sensors, Wang et al. [13] first proposed the full-view coverage, which addresses the issues of viewing direction with the sector sensing model of camera sensor. In a full-view coverage model, a point is full-view-covered if there is always a camera sensor to cover it no matter which direction it faces and the camera sensor’s viewing direction is sufficiently close to the target’s facing direction. Wang et al. further studied the full-view coverage in [14,35,36]. Combining the directional characteristic with high energy consumption of the camera sensor, the authors of [37,38] have been using mobile camera nodes to complete the full-view barrier coverage. Based on the full-view coverage, Ma et al. [39] focused on the minimum camera barrier coverage problem (MCBCP) in wireless camera sensor networks. However, the camera sensor only offered a 2D sensing model in the above works.

Studies on basic barrier coverage types and sensing models are discussed. Most existing works focused on considering target as a point, ignoring extraction the real ROI. Besides, for more precise monitoring tasks, there is resolution requirement to recognize a real object in camera sensor networks. Based on a convex optimization approach, a method of multi-camera deployment for visual coverage of a 3D object surface was proposed in [40]. The authors proposed a new resolution criterion, which simultaneously considered both the distance and the view angle. However, it still processed each point of the module surface iteratively in their works. Hence, in this paper, we propose an effective resolution criterion based on a 3D sensing model of the camera sensor.

To the best our knowledge, the works of Wang et al. [1] and Yu et al. [14] are the most related to our study. Based on the full-view coverage, a camera barrier is constructed by a deterministic deployment strategy along the barrier line in [1]. Furthermore, Yu et al. [14] proposed local face-view barrier coverage, a novel concept that achieves statistical barrier coverage in camera sensor networks leveraging intruders’ trajectory lengths along the barrier and head rotation angles, and also employed the deterministic deployment strategy along the barrier line. Our work is motivated from [1,14], but with two distinct differences. First, the above two concepts take into account the effective angle of the camera sensor’s viewing direction and the intruder’s facing direction. However, both full-view barrier coverage and local face-view barrier coverage only consider the sector sensing model, which is a 2D model. In contrast, we study the 3D sensing model of the camera sensor, which is closer to practical applications. In addition, not only should the effective angle in the horizontal direction be utilized, but also the effective angle in the vertical direction should be taken into account for the real application. For most real-world applications, camera sensors are deployed within a certain height from the ground, which can result in an angle in the vertical direction between the intruder’s facing direction and the viewing direction of one camera sensor. Besides, the intruder’s head can also rotate up and down. Then, we should consider the 3D sensing model of camera sensors for the real barrier coverage.

## 3. 3D Sensing Model Based on ROI

In this section, we first describe preliminaries, and then discuss the 3D sensing model and the realistic resolution criterion of camera sensors.

### 3.1. Preliminaries

**Definition** **1** (Region of interest, ROI). What we are concerned with in the image, or a portion of the target area.

The ROI is significantly different for different monitoring tasks. For example, the color and the license plate number of the car are our concern when we track a car. Similarly, the face is our concern when we monitor a person [41]. Since the ROI in the image usually manifests to rectangular ROI, we will state rectangular ROI in this paper. Besides, the intruder’s face is used as an example to illustrate the ROI.

**Definition** **2** (Effective resolution). The real number of pixels of the ROI in the image plane when the camera sensor captures the ROI.

There are obviously different resolution requirements for different monitoring tasks. A high resolution image is required to identify the details of the ROI. In contrast, the number of pixels if only to identify the target contour will be greatly reduced. For example, when tracking a moving person, the desired resolution is completely different for identifying the color of clothing and the facial expression.

**Definition** **3** (Effective width). The width of the ROI captured by the camera sensor, denoted by $W_{e}$.

**Definition** **4** (Effective length). The length of the ROI captured by the camera sensor, denoted by $L_{e}$.

**Definition** **5** (Effective horizontalangle). The angle between the target’s facing direction and the camera sensor’s viewing direction in the horizontal plane, denoted by α, $\alpha \in \left[0,\frac{\pi }{2}\right)$.

It must be noted that the effective horizontal angle is part of the reason for full-view [1,13] and face-view [14], respectively.

**Definition** **6** (Effective vertical angle). The angle between the target’s facing direction and the camera sensor’s viewing direction in the vertical plane, denoted by β, $\beta \in \left[0,\frac{\pi }{2}\right)$.

### 3.2. 3D Sensing Model

**Figure 2:**
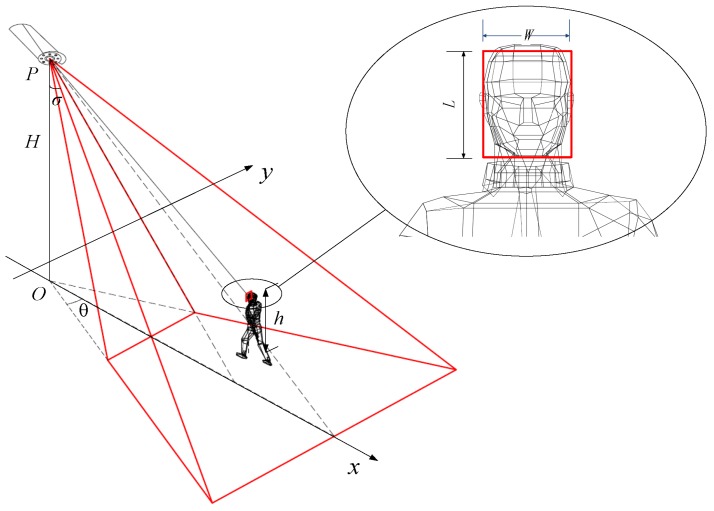
Three-dimensional (3D) sensing model based on region of interest (ROI).

Combined with the 3D sensing model [32], we proposed a 3D sensing model based on ROI. Figure 2 illustrates the 3D sensing model and the ROI.

The 3D sensing model is denoted by 5-tuple $\left(P,\vec{D},\sigma ,\theta ,\gamma \right)$, where P=(x,y,H) is the location of a camera sensor in 3D space. O=(x,y,0) is the location of the camera sensor in the ground, and *H* is the height of the camera sensor from the ground. $\vec{D}=\left(\varphi ,\phi \right)$ is the sensing orientation of the camera sensor. Unless otherwise specified, φ and ϕ are the components along negative direction of *z*-axis and positive direction of *x*-axis, respectively. σ is the minimal value of φ, and θ and γ are the horizontal and vertical one-half angles of the FoV around $\vec{D}$, respectively.

The imaging results of the ROI are completely different using different properties of camera sensors. In this paper, the focal length of camera sensor is denoted by *f*. $r_{u}$ and $r_{v}$ are the number of pixels along the horizontal and vertical directions per unit length in the image plane. In other words, the image size of the ROI on the image plane is l×w. L and W denote the length and width of the ROI, respectively. *D* is the distance between the center of ROI and the camera sensor, and *h* is the height distance between the center of ROI and the ground.

### 3.3. New Resolution Criterion

**Theorem** **1.** *The effective resolution of the ROI captured by the camera sensor is,*

$$\frac{LWr_{u}r_{v}f^{2}}{{\left(H-h+L/2\right)}^{2}}\cos\alpha \cos\beta \sin^{2}\beta$$

**Figure 3:**
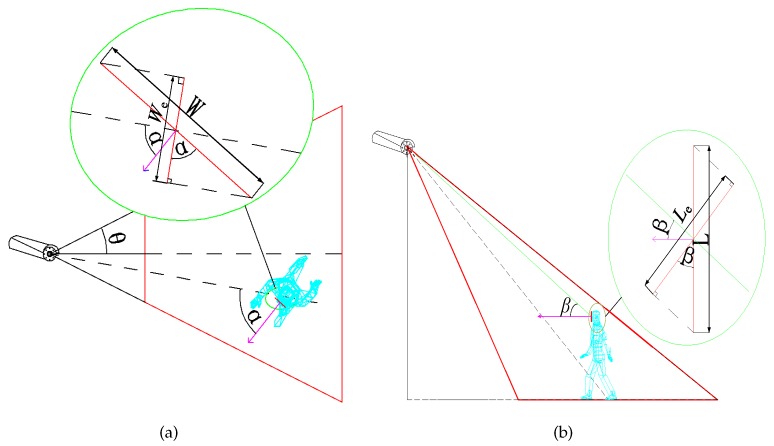
(**a**) The vertical view of 3D sensing model based on ROI; (**b**) The lateral view of 3D sensing model based on ROI.

**Proof** **of** **Theorem** **1.** Figure 3 illustrates the ROI in the horizontal and vertical projections. When the target is moving in the FoV of the camera sensor, effective horizontal angle α and effective vertical angle β are formed between the target and the camera sensor. ☐

Effective width is,

(1) $$W_{e}=W\cos\alpha$$

Effective length is,

(2) $$L_{e}=L\cos\beta$$

As we know, the optical imaging principle is,

(3) $$\frac{L_{e}}{l}=\frac{D}{f}$$

(4) $$\frac{W_{e}}{w}=\frac{D}{f}$$

From Equations (3) and (4), we can get the size of ROI on the image plane, which is,

(5) $$lw=\frac{L_{e}W_{e}f^{2}}{D^{2}}$$

According to Equations (1) and (2), the size of ROI on the image plane is,

(6) $$lw=\frac{LW\cos\alpha \cos\beta f^{2}}{D^{2}}$$

The effective resolution of the ROI is,

(7) $$R_{e}=lwr_{u}r_{v}=\frac{LW\cos\alpha \cos\beta r_{u}r_{v}f^{2}}{D^{2}}$$

Figure 3b shows,

(8) $$\sin\beta =\frac{H-h+L/2}{D}$$

*H* is the height of the camera sensor from the ground and *h* is the height distance between the center of ROI and the ground. The length of the ROI is *L*.

According to Equations (7) and (8), we can get the effective resolution of the ROI, which is,

(9) $$R_{e}=\frac{LWr_{u}r_{v}f^{2}}{{\left(H-h+L/2\right)}^{2}}\cos\alpha \cos\beta \sin^{2}\beta$$

With this new resolution criterion, we can state one visual coverage constraint using a prescribed threshold $R_{t}\in R^{+}$ as the task parameter,

(10) $$R_{e}\geq R_{t}$$

## 4. Barrier Coverage

In this section, we first present the barrier coverage using the 3D sensing model based on ROI. Next we describe the width of barrier coverage and the distance between two adjacent camera sensors. Finally, we introduce the coverage probability of barrier coverage with respect to different tasks.

### 4.1. Deployment Pattern

**Figure 4:**
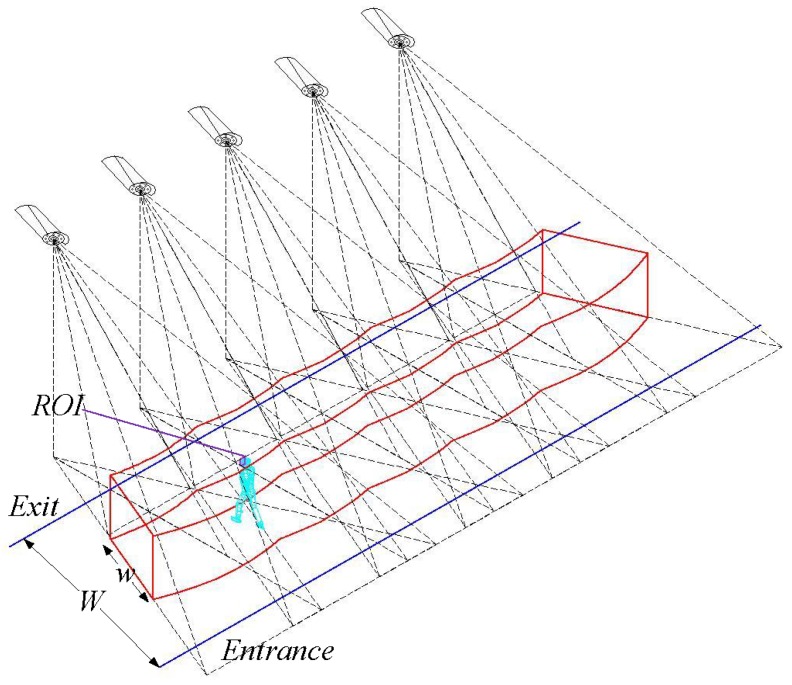
Barrier coverage using the 3D sensing model.

**Figure 5:**
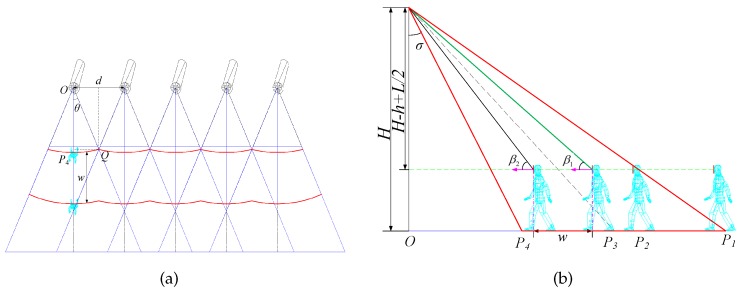
(**a**) The vertical view of barrier coverage; (**b**) The lateral view of barrier coverage.

We consider a regular pattern [14] in which all camera sensors are deployed on a line along the exit and all camera sensors face toward the entrance, and the width between the entrance and the exit is *W*. All camera sensors have the same height and the distance of every two adjacent camera sensors is equivalent. For this deployment pattern, we can achieve a 3D barrier space with width *w* that needs to be monitored. As a result, we obtain the barrier coverage as shown by the ’red space’ in Figure 4. Figure 5a,b are the vertical view of barrier coverage and the lateral view of barrier coverage, respectively.

In this paper, we consider a person to illustrate the intruder crossing the barrier area. When the intruder sneaks into the barrier area, the barrier coverage should effective capture the intruder’s face while meeting the requirement of resolution. Then the task is to detect and capture the intruder’s face (or the ROI).

### 4.2. Width of Barrier Coverage

Before discussing the width of barrier coverage, we give some definitions from our new resolution criterion.

As seen in Figure 5b, one foot enters the FoVs of camera sensors when the intruder’s position is $P_{1}$. However, the intruder’s face which is what we are concerned about is still not observed. In other words, the ROI is the intruder’s face. Hence, the person needs to move to $P_{2}$, where $\beta =\frac{\pi }{2}-\left(\sigma +2\gamma \right)$. However, the ROI may also not meet the requirement of high resolution when the person (including the person’s face) is within the camera sensors’ FoVs.

**Definition** **7** (Minimum effective vertical angle). The minimum effective vertical angle $\beta_{1}$ is that the minimum value of effective vertical angle meets the required resolution when the target is moving in the FoV of camera sensor.

As seen in Figure 3b, when the person is located at $P_{2}$, the resolution may not meet the requirement of the monitoring task. When the person arrives at $P_{3}$, the resolution satisfies the requirement, and the effective vertical angle is $\beta_{1}$.

**Definition** **8** (Maximum effective vertical angle). The maximum effective vertical angle $\beta_{2}$ is that the maximum value of effective vertical angle meets the required resolution when the target is moving in the FoV of camera sensor.

The person continues to move to $P_{4}$, the effective vertical angle is $\beta_{2}$ ($\beta_{2}\leq \frac{\pi }{2}-\sigma$). When the person continues to move forward, the effective vertical angle will be larger. However, we can easily prove that the resolution of ROI captured by camera sensors will decrease (see Equation (9)).

The effective vertical angle changes from $\beta_{1}$ to $\beta_{2}$, according to that the position of the target changes from $P_{3}$ to $P_{4}$, as shown in Figure 5b. We can get $w=\left|OP_{3}\right|-\left|OP_{4}\right|$.

Thus, the width of barrier coverage meeting the task requirement of resolution is,

(11) $$w=\left(H-h+\frac{L}{2}\right)\left(\cot\beta_{1}-\cot\beta_{2}\right)$$

Note that the width of barrier coverage from Equation (11) does not consider the effective horizontal angle α. In most cases, the ROI and the camera sensor cannot hold on the optimal effective horizontal angle α=0, since the intruder’s walking trajectory changes with time. In other words, only in the barrier area with width *w* can we discuss the full-view coverage in camera sensor networks. This is an important difference from previous works.

### 4.3. Distance Between Two Adjacent Camera Sensors

The maximum of the effective resolution can be obtained when the ROI and the camera sensor hold on the optimal effective horizontal angle α=0. As is shown in Figure 5a, $\left|OQ\right|=\left|OP_{4}\right|$.

Then, we have,

(12) $$\left|OQ\right|=\left(H-h+\frac{L}{2}\right)\cot\beta_{2}$$

The distance between two adjacent camera sensors is,

(13) $$d=2\left|OQ\right|\sin\theta$$

Then,

(14) $$d=2\left(H-h+\frac{L}{2}\right)\sin\theta \cot\beta_{2}$$

### 4.4. Coverage Probability

Once an intruder comes into the barrier area, camera sensors will follow a probabilistic model to capture the intruder. Since the intruder’s trajectory is arbitrary, the resolution of ROI will change with different effective horizontal angles and different effective vertical angles.

**Theorem** **2.** *The maximum effective resolution based on ROI is,*

$$\frac{2\sqrt{3}}{9}\frac{LWr_{u}r_{v}f^{2}}{{\left(H-h+L/2\right)}^{2}}$$

**Proof** **of** **Theorem** **2.** When a target is moving in the barrier area, the ROI of the target is captured by camera sensors. We can get the effective width and the effective length from Equations (1) and (2). Since $W_{e}\geq 0$ and $L_{e}\geq 0$, the ranges of the effective horizontal angle α and the effective vertical angle β are both $\left[0,\frac{\pi }{2}\right]$. When α=0 and $\beta =\arcsin\frac{\sqrt{6}}{3}$, cosα gets the maximum value 1 and $\cos\beta \sin^{2}\beta$ gets the maximum value $\frac{2\sqrt{3}}{9}$. Besides, from Equation (9), camera sensors in the barrier can achieve the maximum value $R_{\max}=\frac{2\sqrt{3}}{9}\frac{LWr_{u}r_{v}f^{2}}{{\left(H-h+L/2\right)}^{2}}$. In other words, the optimal effective horizontal angle is α=0 and the optimal effective vertical angle is $\beta_{b}=\arcsin\frac{\sqrt{6}}{3}$. ☐

We set $R_{t}$ as the desired resolution of the monitoring task. When $R_{t}\leq R_{\max}$, we can get the coverage probability,

(15) $$p=\frac{R_{e}}{R_{\max}}$$

As a result, we have,

(16) $$p=\left\{\begin{array}{cc} \frac{3\sqrt{3}}{2}\cos\alpha \cos\beta \sin^{2}\beta & R_{t}\leq R_{\max}\\ 0 & R_{t}>R_{\max} \end{array}\right.$$

From Equation (16), we find that the coverage probability is only related to α and β. α and β are the effective horizontal angle and the effective vertical angle between the camera sensor’s viewing direction and the target’s facing direction, respectively.

The resolution can be easily obtained by the coverage probability multiplies maximum effective resolution. Then, compared the computed result with the desired resolution of the monitoring task, our barrier coverage was successfully constructed with a 3D sensing model of camera sensors.

## 5. Evaluation

In this section, we evaluate barrier coverage with the 3D sensing model via simulations. Our simulations first examine the barrier width with respect to different task requirement of resolution. Next, we examine the number of camera sensors per 100 m and the coverage probability with different parameters. Finally, we compare the number of camera sensors needed for barrier coverage with other barrier coverage strategies.

The parameters of camera sensors are H=7 m, f=12 mm, $\sigma =\frac{\pi }{24}$, $\gamma =\frac{\pi }{6}$, $r_{u}=100$ pixles/mm and $r_{v}=100$ pixles/mm. We consider that the target passing through the barrier area is a person, and the ROI is the person’s face. The height of target is h=1.7 m. The parameters of the ROI are L=0.2 m and W=0.15 m.

### 5.1. Barrier Width

**Figure 6:**
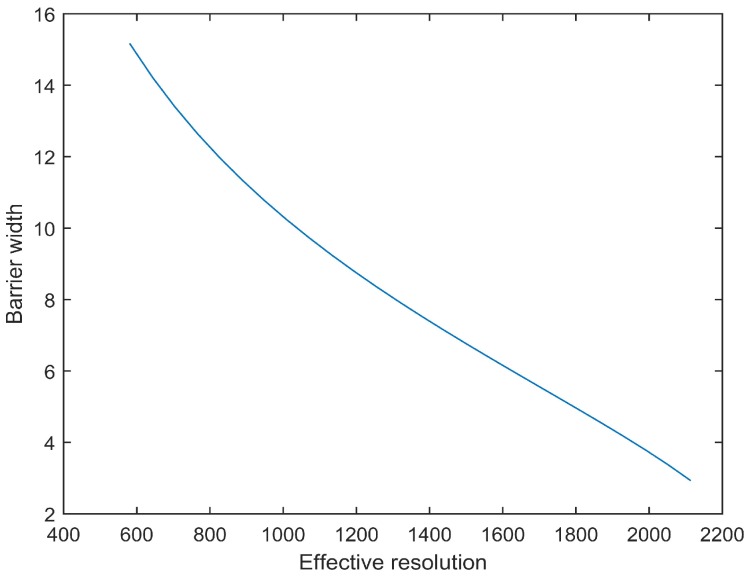
Barrier width vs. required resolution.

We examine the barrier width $w_{e}$ with respect to the required resolution $R_{t}$. The simulation results are shown in Figure 6. For more required resolution, we observe that the barrier width $w_{e}$ decreases quickly as $\theta =\frac{\pi }{6}$. The simulation results are encouraging as the barrier width $w_{e}$ can be determined by the task requirement of the resolution $R_{t}$.

### 5.2. Number of Camera Sensors

**Figure 7:**
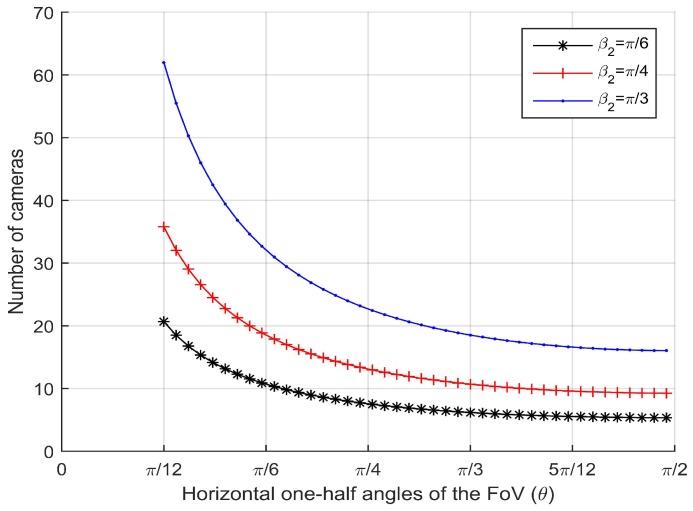
Number of camera sensors per 100 m vs. horizontal one-half angle of field of view (FoV) for different maximum effective vertical angles.

First we evaluate the number of camera sensors per 100 m needed for coverage with respect to the horizontal one-half angle of FoV θ given the maximum effective vertical angles $\beta_{2}=\frac{\pi }{6}$, $\beta_{2}=\frac{\pi }{4}$ and $\beta_{2}=\frac{\pi }{3}$. The simulation results are shown in Figure 7. For the horizontal one-half angle of FoV θ, we observe that the required number of camera sensors decreases rapidly. However, for the horizontal one-half angle of FoV $\theta >\frac{\pi }{3}$, the required number of camera sensors decreases slowly. Actually, when the one-half angle of FoV is close to $\frac{\pi }{2}$, camera sensors become fish-eye camera sensors.

**Figure 8:**
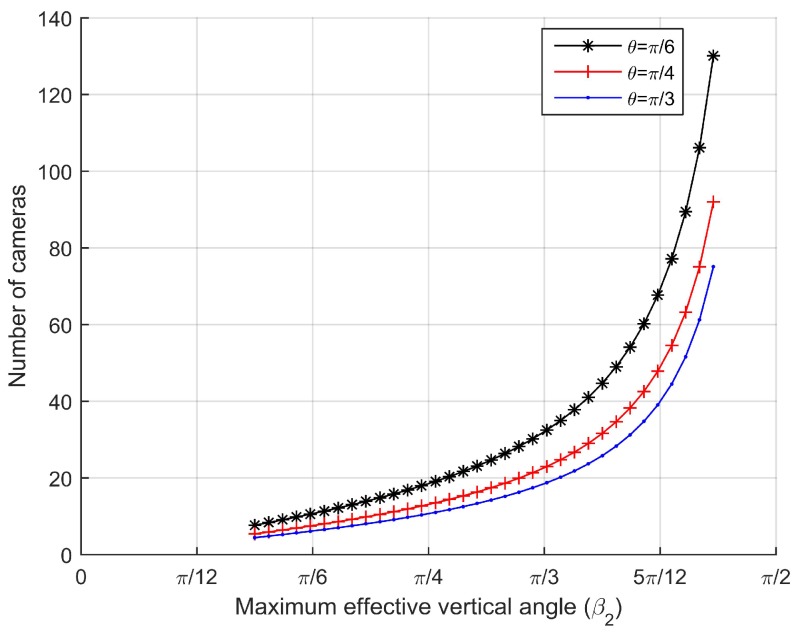
Number of camera sensors per 100 m vs. maximum effective vertical angle for different horizontal one-half angle of FoV.

Next we evaluate the number of camera sensors per 100 m needed for coverage with respect to the maximum effective vertical angle $\beta_{2}$ given the horizontal one-half angles of FoV $\theta =\frac{\pi }{6}$, $\theta =\frac{\pi }{4}$ and $\theta =\frac{\pi }{3}$. By comparing the three curves in Figure 8, it can be seen that as the maximum effective vertical angle $\beta_{2}$ becomes larger, the number of camera sensors needed is also increased. However, since the existing of the effective vertical angle $\beta_{2}$ cannot be 0, the number of camera sensors will obtain the minimum value when the maximum effective vertical angle approaches to the minimum effective vertical angle $\beta_{1}$.

### 5.3. Coverage Probability

**Figure 9:**
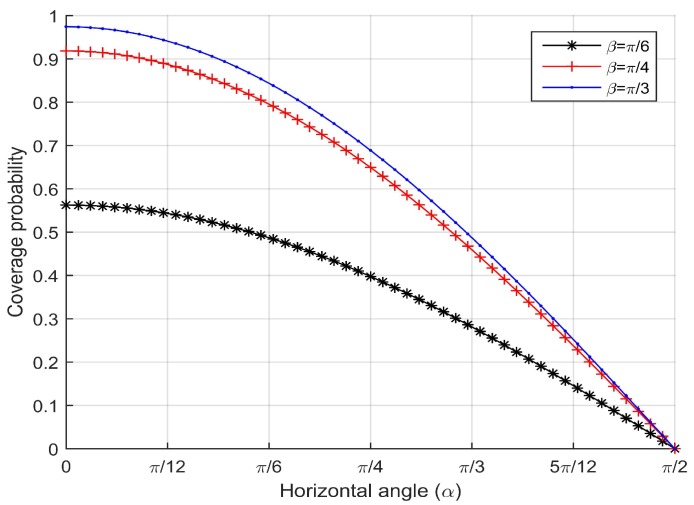
Coverage probability vs. the effective horizontal angle for different effective vertical angles.

First, we examine the coverage probability *p* with respect to the effective horizontal angle α given the effective vertical angles $\beta =\frac{\pi }{6}$, $\beta =\frac{\pi }{4}$ and $\beta =\frac{\pi }{3}$. The horizontal one-half angle of FoV is $\theta =\frac{\pi }{6}$. The simulation results are shown in Figure 9. We observe that as the horizontal angle becomes larger, the coverage probability *p* decreases quickly. When the effective horizontal angle is the optimal effective horizontal angle α=0, the coverage probability *p* becomes the maximum value.

**Figure 10:**
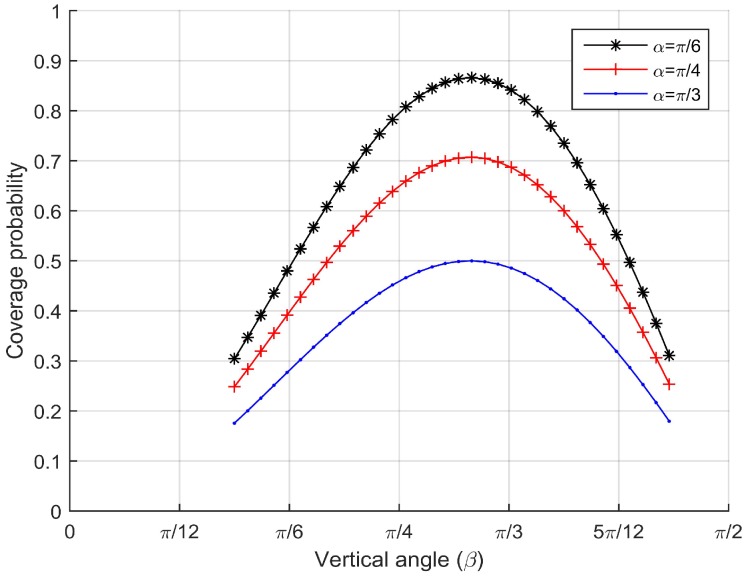
Coverage probability vs. the effective vertical angle for different effective horizontal angles.

Next, we examine the coverage probability *p* with respect to the effective vertical angle β given the effective horizontal angles $\alpha =\frac{\pi }{6}$, $\alpha =\frac{\pi }{4}$ and $\alpha =\frac{\pi }{3}$. We also set the horizontal one-half angle of FoV $\theta =\frac{\pi }{6}$. The simulation results are shown in Figure 10. As we can see, the coverage probability *p* does not change monotonically with the effective vertical angle β. When the vertical angle is the optimal effective vertical angle $\beta_{b}=\arcsin\frac{\sqrt{6}}{3}$, the coverage probability becomes the maximum value.

### 5.4. Comparison

Finally, we compare the number of camera sensors per 100 m required with local face-view barrier coverage [14] and full-view barrier coverage [1].

**Figure 11:**
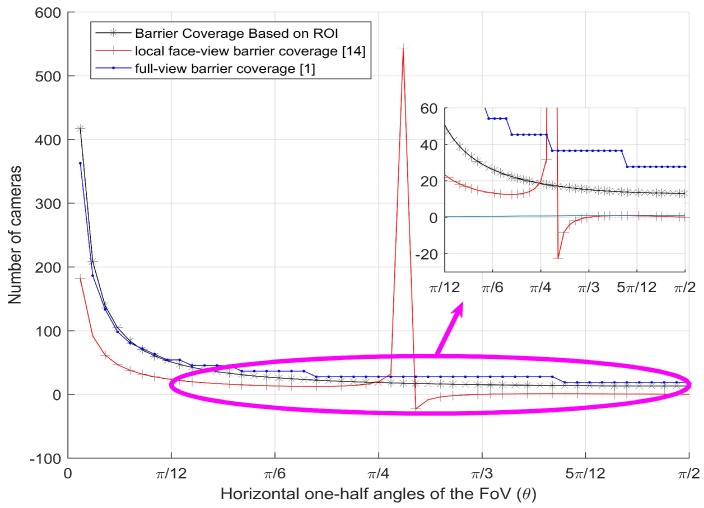
Number of camera sensors per 100 m vs. the effective horizontal angle for different barrier coverage strategies.

We set the coverage probability 1, and the other parameters with the ones used in local face-view barrier coverage and full-view barrier coverage. As shown in Figure 11, as the horizontal one-half angle of FoV θ becomes larger, the number of camera sensors required of our barrier coverage and full-view barrier coverage strategies both decrease, but our barrier coverage needs much fewer camera sensors than the full-view barrier coverage. Although the number of camera sensors required of our strategy is a little more than the local face-view barrier coverage when θ is smaller, the local face-view barrier coverage will be unreasonable for all angles of FoV θ.

The authors [14] only consider one effective angle in the horizontal direction. In contrast, we simultaneously consider two effective angles in the horizontal and vertical directions. Thus, we need more camera sensors to construct the 3D barrier coverage of camera sensor networks. Furthermore, our barrier coverage is a 3D barrier coverage, and it is closer to the real life.

## 6. Discussion

In this section, we analyze the robustness of our barrier coverage and discuss how to ensure the coverage probability with our deployment strategy.

**Figure 12:**
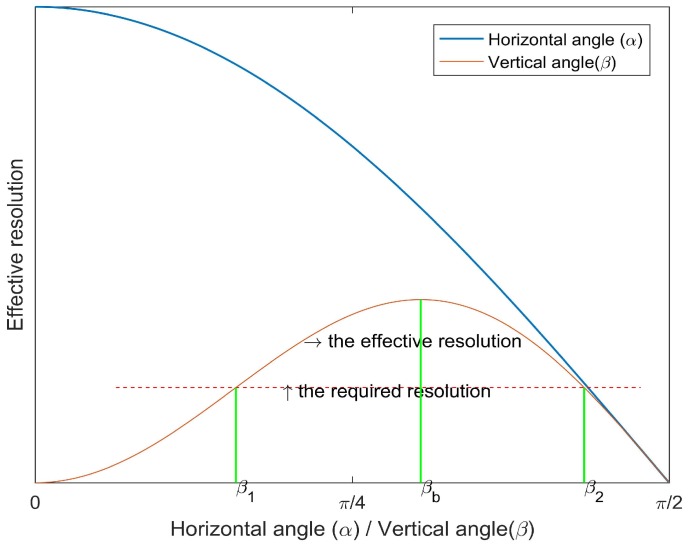
The effective resolution based on the ROI vs. horizontal angle (or vertical angle).

Our barrier coverage can not only capture the intruder that attempts to cross the barrier region, but also recognizes who is the intruder. As we mentioned above, to recognize an intruder, the resolution of the ROI captured by camera sensors should greater than the desired resolution $R_{t}$ of the monitoring task. In other words, the effective resolution of the ROI should satisfy $R_{e}\geq R_{t}$. Since the camera sensors are deployed with a height from the ground, it is hard to keep stealthy for a sophisticated intruder (e.g., the intruder’s head can rotate down to escape from the cameras). However, the deployed cameras can capture and recognize the intruder successfully if the sophisticated intruder’s head keeps in a reasonable angle range in horizontal and vertical directions. As shown in Figure 12, if the effective horizontal angle meets α≥0 and the effective vertical angle satisfies $\beta_{1}\leq \beta \leq \beta_{2}$, we will have the change to capture and recognize the intruder with our deployment strategy.

**Figure 13:**
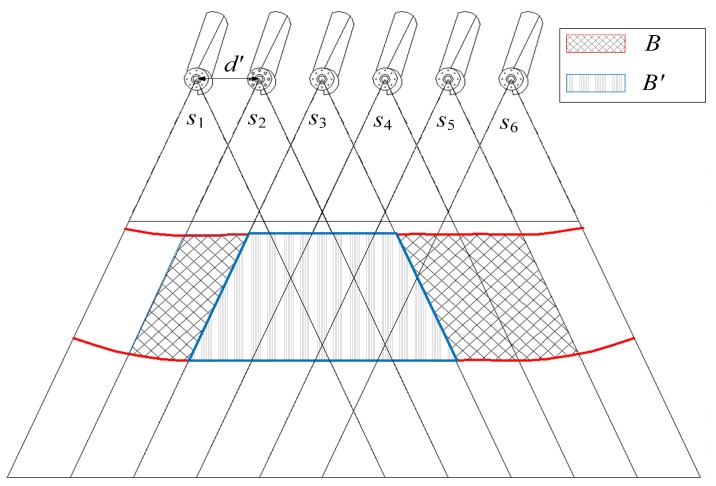
An example of two-barrier coverage for camera sensor networks.

It is important for barrier coverage to capture all the intruders that attempt to cross the barrier region. However, it is a challenge for the barrier to capture and recognize every intruder. From the above analysis, given the camera’s parameters and the monitoring task, we find that the coverage probability is related to the horizontal and vertical angles between the camera sensors and the ROI. In some harsh environmental conditions, the challenge is how to deploy camera sensors to hold with coverage probability p=1 even when a camera is damaged (e.g., due to hardware failure). In this worst case scenario, we can refer to the strategy of K-barrier coverage [7,30] to construct fault tolerance barrier coverage. In our deployment strategy, we should shorten the distance between two adjacent camera sensors to construct K-barrier coverage. As depicted in Figure 13, to enhance the fault tolerance of the camera sensor networks, the distance between two adjacent camera sensors satisfies $d^{\prime }<d$, and a set $S=\{s_{1},s_{2},\cdots ,s_{6}\}$ of camera sensors are deployed to form two-barrier coverage *B*. Thus, once an intruder attempts to cross the barrier region, at least two cameras will capture him. For example, if the camera sensor $s_{3}$ malfunctions, the monitoring area $B^{\prime }$ is also be captured by other cameras and guarantees the coverage probability.

## 7. Conclusions

This paper presented a new resolution criterion based on 3D sensing model of camera sensors. Unlike the traditional sensing models where the target was considered as a point in the sensing area, our sensing model added more realistic element (e.g., ROI). In this sensing model, the effective horizontal angle and the effective vertical angle between the camera sensor and the ROI, as well as characteristics of camera sensor and target (e.g., resolution of the camera sensor, the height of camera sensor from ground and the size of the ROI) were the major factors. Through the analysis of the sensing model, we obtained the maximum effective resolution of the ROI. Based on the proposed 3D sensing model, we have established barrier coverage. We studied the width of barrier coverage, the distance between camera sensors and the coverage probability. The simulation results validated the number of required camera sensors is more reasonable in the real world. To the best of our knowledge, this is the first work on barrier coverage by means of a 3D sensing model of a camera sensor.
